# A mixed-methods analysis of the role of online social support to promote psychological wellbeing in new mothers

**DOI:** 10.1177/20552076221147433

**Published:** 2023-01-04

**Authors:** Sally Henton, Vivien Swanson

**Affiliations:** Psychology Division, School of Natural Sciences, 7622 University of Stirling, Stirling, UK

**Keywords:** maternal health, social media, social support, perinatal wellbeing, maternal stress, parental stress, motherhood, perinatal coping

## Abstract

**Objective:**

Perinatal mental health problems affect between 1 in 3 and 1 in 10 women globally. Using social media could offer helpful support to new mothers to mitigate this. This research examines the impact of online social support on parental stress, and the mediating effect of maternal wellbeing. The goal is to improve understanding of how to optimise online maternal support to improve anxiety and reduce long-term stress for mother and child.

**Design:**

A mixed-methods, convergent parallel design (QUANT-QUAL) is adopted to facilitate examination of the complex association between constructs.

**Methods:**

A Qualtrics online survey was administered via social media to mothers of children under two (n = 151). Quantitative multiple regression analyses assessed perceptions of online social support overall and in separate domains (Social Networking Sites Usage and Needs Scale) as a predictor of parental stress (Parental Stress Scale) and the potential mediation effect of mental wellbeing (Short Warwick–Edinburgh Mental Wellbeing Scale). Purposely designed survey open-text questions allowed participants to describe the detail and impact of online support experiences and common stresses and formed the basis of a qualitative reflexive thematic analysis examining online support and maternal mental health.

**Results:**

Mixed-method findings indicate that mothers perceiving more value in online support have higher stress levels and lower wellbeing than others. Mental wellbeing was a partial mediator of the relationship between online support and parental stress. Non-significant statistical effects were reinforced by qualitative themes indicating online support provided safe guidance, peer solidarity and parenting escape.

**Conclusions:**

Maternal online support was predominantly used to cope with high stress, explaining positive stress correlations. Statistically, online coping strategies contributed little to mental wellbeing. Nevertheless, online support was regarded as a valuable and reassuring tool by some participants. Health professionals could improve perinatal anxiety coping by facilitating quality online support networks.

## Background

World Health Organisation (WHO) data indicates that 1 in 10 perinatal women (spanning pregnancy and the first year after birth) within developed countries, experience depression or anxiety, rising to levels as high as 1 in 3 in developing countries.^1^ WHO's resulting global strategic goal to improve maternal mental health is driven by its associations with maternal mortality and morbidity, as well as adverse implications for child development.^2^ Problematically, the commonality between early motherhood issues and mental health symptoms such as fatigue leads to under-diagnosis.^1^

Within the United Kingdom, one in five women experience perinatal mental health issues.^3^ Additionally, research identifies many new mothers as being poorly prepared for motherhood and under-supported,^4^ with the enduring stigma surrounding maternal mental health limiting potential help-seeking.^5^ Women with high stress and low-level anxiety therefore often remain unsupported. In a recent UK survey of 2300 mothers, half experienced anxiety, yet only 7% were referred to specialist care.^6^ There is value in identifying mechanisms to support women with stress or anxiety before problems escalate.

Furthermore, the recent COVID-19 pandemic has exacerbated psychological distress. A UK-based survey conducted with 5474 mothers (pregnant, or with children aged under 2) during COVID-19, found that 87% were more anxious and 67% felt less able to cope during lockdown restrictions.^7^ Similarly, a Canadian survey found perinatal mothers with scores indicative of depression in 15% of pre-pandemic responses and 40% of pandemic responses.^8^ Opportunities to facilitate improvements in maternal mental wellbeing should therefore be explored.

### Mental wellbeing

Despite the maternal research focus on anxiety and depression, modern understanding of mental wellbeing goes beyond the absence of mental illness, for example, depression, to span life satisfaction, psychological function and growth. Traditional multi-faceted definitions combine hedonic wellbeing aspects focusing on positive affect and enjoyment, with eudaimonic wellbeing aspects which focus on psychological function, incorporating the ability to effectively cope with daily stresses, together with individual growth through the realisation of one's purpose and contribution. ^9^

New motherhood is associated with both positive and negative psychological experiences likely to involve complex life change,^10^ with some evidence demonstrating associations between new motherhood and a continual decline in mental wellbeing up to 7 years after birth.^11^ Opportunities to reduce maternal transitional pressures and optimise wellbeing, therefore, warrant exploration.

### Parental stress

The multi-farious and inter-weaving stress definitions identify both external stressors, from daily hassles to life changing events, prompting physiological responses and psychological appraisal, as well as stress as an impactful internal emotion characterised by feelings of pressure and anxiety, which can in turn influence physiological health outcomes.^12^ The coping resources that an individual draws on in response to appraised stress are central to situational outcomes.^12^ Moreover, the heterogeneity within stress research highlights the dynamic and complex nature of stress and the associated difficulty of examining individual effects.^13^

Parenting becomes a source of stress when tension develops between role demands and individual resources or expectations.^14^ The early years of motherhood involve complex identity and behavioural norm changes^10^ all of which could trigger such negative self-evaluation. Maternal stress could be considered a particularly important sub-category of parental stress with research highlighting adverse impacts for mothers, for example, poorer nutrition and exercise^15^; as well as on children, for example, reduced breastfeeding^16^ and insecure attachment.^17^

Maternal pressure is operationalised by Berry and Jones^18^ in an examination of the rewards and demands of parenting and the bi-directional synchrony of the parent and child relationship.^19^ Here a mother's stress negatively impacts her caregiving quality, which can increase child insecurity, distress and anger, which in turn heightens parental stress. Saisto et al.,^20^ for example, found parental stress correlated with multiple variables, predicting stress levels up to 3 years after birth. Opportunities to minimise parental stress are therefore worth investigating. However, parental stress research predominantly focuses on parents facing particularly stressful circumstances, for example, parents of autistic children,^14^ suggesting a more general maternal focus is warranted.

### Social support

Social support is a dominant, multi-faceted, stress-coping resource whereby psychological emotion-focused and tangible material strength is provided through interactions between an individual and their close network, enabling coping and growth.^21^ The long-standing link between social support and mental health is complex, creating multiple theories for associations.^22^ Research demonstrates positive links between social support and mental health.^23^ In particular, low social support levels are frequently associated with an increased risk of depression, and high social support levels linked to increases in positive self-esteem and mood, as well as a reduction in distress.^23^ Further complexity stems from the sub-categorisation of social support functions.^22^ Perceived emotional support is regarded as particularly important for mental wellbeing and relates to the extent a person feels part of a caring network.^23^

Research also demonstrates positive effects of social support on maternal self-esteem, life satisfaction and stress.^24^ Theoretically, mothers with high levels of social support feel more able to cope with perceived stress, for example, by using social support networks as an emotion-focused strategy. Vaezi et al.,^25^ for example, found mothers with higher levels of social support were less likely to develop postpartum depression. In particular, empathic social support from peers becomes especially valuable to offset perinatal attempts to downplay coping struggles.^5^

Online communication using social networking sites (SNS) is emerging as an increasingly important and integrated component of social support, enabling additional and remote means for individuals to socially connect with others.^26^ Parents can be particularly reliant on this web-based social connection using SNS such as Facebook and Instagram to interact and network.^27^ Notably, Ginja et al.^28^ found a positive correlation between social support and mental wellbeing but no significant effect of the technology use, with the pervasiveness of technology use limiting analysis. Given the extent of maternal SNS use, it is important to develop understandings of construct associations, functions and impacts.

The complex bi-directional relationship between online support and mental wellbeing can either be protective or exacerbating, largely dependent on the quality of interactions.^27^ Higher affect results, for example, when SNS use is motivated by active support seeking as opposed to passive browsing.^29^ Wang et al.^30^ found a negative correlation between wellbeing and passive SNS use, with SNS activity characterised by a need to escape feelings or reinforce personal behaviours, but lacking protective social support elements.

Existing research on SNS use is predominantly qualitative, and focused on eliciting maternal experiences, for example, perinatal sources of anxiety and support.^4^ In contrast, quantitative studies largely focus on specific parenting aspects,^31^ or usage analyses.^32^ Detailed exploration of online social support's perceived value could enable SNS use to be proactively harnessed to improve coping and anxiety levels of new mothers.

This study aims to examine use of the online social support potential of SNS in mothers with children under 2 and its impact on parental stress. The study will also investigate the mediating effect of maternal mental wellbeing as a possible explanatory factor, to understand how wellbeing influences stress and online support.

## Methods

### Research design

This mixed-methods design adopted a fixed, concurrent triangulation approach to maximise construct understanding.^33^ Quantitative and qualitative (QUANT–QUAL) data were collected concurrently within a one-phase survey. Sequential analysis began with the quantitative data before progressing to an equally weighted qualitative analysis. Findings were then examined and interpreted together. The approach rationale is that diverse methods are complementary, providing rich thematic insights and integrating with statistical findings.^34^

### Participants and procedure

The study was granted ethical approval by The University of Stirling General Ethics Committee. Ethics approval reference: GUEP 2021 2270 1751. Eligible participants were mothers with a child aged 2 or under, with no recent diagnosis of depression. This ensured that participants were sufficiently psychologically robust to consider questions relating to their mental wellbeing, without adverse consequences. Power calculations based on a medium effect size at 80% power (Zhao et al.,^14^ r = 0.314), created a target sample size of 100 participants, allowing for missing data.

The recruitment strategy was purposive and strategic. Local groups with a maternal client base and an active online presence were contacted regarding advertising (Appendix A). Additionally, snowball sampling broadened recruitment reach through personal social media accounts.

The advertisement included an explanation of the research purpose, the participant criteria and a link to the online survey. The survey remained live for 4 weeks during May 2021. At this point Scotland was in month 14 of the COVID-19 pandemic and was moving to Phase 2 of a route map aimed at a gradual lifting of lockdown restrictions. An online survey (Appendix B) was chosen to maximise participant reach and perspective diversity whilst affording participants anonymity when discussing potentially sensitive topics. The 15-minute survey was hosted on Qualtrics software (version: July 2021). Online informed participant consent was sought before survey initiation. The online survey was first piloted using convenience sampling with five volunteers fitting the inclusion criteria to assess survey usability.

### Quantitative measures

The main study outcome was parental stress, with online social support as the predictor and mental wellbeing as a potential mediator. Three scales were combined within one survey instrument to balance brevity with rich construct understanding.^35^

#### Parental stress

Berry and Jones's (1995) Parental Stress Scale (PSS)^15,18^ was used, chosen as a parent-specific stress scale that facilitated insights about both the demands and rewards of parenting. This 18-item questionnaire asks parents to rate their agreement with statements, for example, ‘*I enjoy spending time with my child(ren)*’. The measure covers the rewards and demands of parenting, with response options using a five-point Likert scale (1 = strongly disagree, 2 = disagree, 3 = undecided, 4 = agree and 5 = strongly agree). The scores are calculated by summing responses, with positive statements reverse scored (1, 2, 5, 6, 7, 8, 17 and 19) and higher scores representing greater parental stress. Possible scores range from 18 to 90. Reliability was 0.83 using Cronbach's alpha.^18^ A recent systematic review of 25 studies found the measure to be flexible and effective for a diverse range of populations with minimal adaptation.^19^

#### Online social support

Ali et al.'s^36^ Social Networking Sites Usage and Needs (SNSUN) scale was adopted to measure online social support, chosen for its breadth of survey components that go beyond simple social networking usage information to understand participant needs from SNS. Frequency questions outline participant ‘usage’ of online support by covering *how often and how many listed SNS are used, and how much time is spent on SNS's per day.* Two questions were removed from the published scale based on pilot feedback.

SNS ‘needs’ outline participant perceived value of online support, and are sub-divided into five domains: diversion, cognitive, affective, personal and social. Twenty statements (four for each domain), for example, ‘*SNS's help me feel less lonely*’ are measured using a five-point Likert scale (1 = strongly disagree, 2 = disagree, 3 = undecided, 4 = agree and 5 = strongly agree). Scores are summed, high scores indicating higher SNS needs. Three statements had minor wording amendments to make them applicable to the maternal context. Possible scoring ranged from 0 to 100. Scale reliability was 0.92 using Cronbach's alpha, with strong correlation within and between dimensions and applicability across diverse populations.^36^

#### Mental wellbeing

The Short Warwick–Edinburgh Mental Wellbeing Scale (SWEMWBS) (© NHS Health Scotland, Universities of Warwick and Edinburgh, 2008)^37^ measured mental wellbeing, chosen as a brief scale that facilitated rich participant insights spanning hedonic and eudaimonic aspects of mental wellbeing. This seven-item scale, incorporates hedonic and eudaimonic statements, with an emphasis on functioning.^38^ Positively worded statements relating to the past 2 weeks, for example, ‘*I've been feeling useful*’ are rated using a Likert scale (none of the time (1), rarely (2), some of the time (3), often (4) and all of the time (5)). Scores are combined to create a total raw score, converted within Microsoft Excel using a metrics conversion table provided to enable comparison between studies, with high scores representing greater mental wellbeing.^38^ Possible scoring ranged from 7 to 35.

The internal consistency reliability of the SWEMWBS was 0.84 using the Person Separation Index (R),^37^ which is regarded as a more conservative estimate than the Cronbach's alpha measurements used by the SNSUN and PSS.^39^ The SWEMWBS has a high correlation with the longer WEMWBS (0.95), low social desirability bias and is cross-culturally validated.^38^

### Qualitative data collection

Open-text questions asked participants to provide examples of common online interactions, their usefulness, and any impact of COVID-19 on online activity. Participants were also asked to describe common stresses and their impact on daily life. Risks of participant attrition and response brevity^35^ were targeted by making all questions optional, utilising an essay style box with maximum character limits (20,000) and explicitly encouraging open-question response detail and examples through question wording. Downloaded Qualtrics responses (Qualtrics, Provo UT) were held anonymously at participant id/question number level within Microsoft Excel.

### Quantitative data analysis

Statistical analyses were conducted within R (v1.3.1093).^40^ The alpha level used as the significance criterion for statistical tests was *p* < 0.05. Assumptions of linearity, independence of observations, normality and homoscedasticity were tested using the *gvlma* function^41^ and identified one outlier (PSS = 80, ID: 590931). Analyses were run with and without the outlier, and the overall pattern of data remained unchanged. Given that the outlier is a genuine response, results are reported with the outlier included.

Phase 1 involved generating descriptive statistics for all demographic and social networking usage data. Phase 2 used the Pearson correlation coefficient (*cor* function)^42^ to assess variable relationships. Phase 3 assessed causal mediation through multiple linear regression analyses (*lm* function) and via bootstrapping (*mediate* function),^43^ with online social support (SNS needs) as the predictor, maternal wellbeing as the mediator and parental stress as the outcome variable. Phase 4 repeated Phase 3 at SNSUN sub-domain level. All predictor variables were mean-centered for Phases 3 and 4 to reduce multi-collinearity risk.^44^

### Qualitative data analysis

A reflexive thematic analysis^45^ was conducted on open-question survey responses using a critical realist approach, which acknowledges the influence of both the social world and underlying causal mechanisms.^46^ As a parent of two young children who had utilised social networking groups for early years support, researcher one's (SH) position both within and outside the study topic created a perspectival closeness that aided analytical insight but demanded reflective scrutiny surrounding thematic influences.^47^ Researcher two's (VS) external position with regard to parenting social network use minimised potential bias, and her broader maternal health field expertise provided an additional beneficial layer to analytical interpretations.

The six-phase reflexive and cyclical process^45,47^ began with familiarisation. Data was considered as one corpus to better elicit rich understanding. The second, code generation step utilised an inductive approach to systematically explore participant perspectives.^48^ The research question was used to narrow the scope towards how online support is used by mothers to reduce stress and influence maternal wellbeing.

Given anticipated response brevity, data interpretation was conducted at a predominantly semantic level, identifying meaning based on explicit patterns evident within the data.^45^ The third, theme construction step involved re-arranging data into clusters of meaning and developing themes reflective of the overall data story.^45^ The remaining steps, revising themes, defining themes and producing the report involved clarifying and refining understanding with the aid of thematic mapping.^46^

### Data analysis integration

Following sequential analysis of quantitative and qualitative results, the concurrent triangulation approach integrated findings. Significant quantitative results were considered in parallel with qualitative thematic insights to determine possible interpretations and explanations.

## Quantitative results

### Phase 1: Descriptive statistics

#### Sample demographics

Out of 261 who started, 151 participants completed the survey. A survey was excluded where quantitative scales were <90% complete as this would have confounded total scoring. Most women were from the United Kingdom, aged 31 to 35 years (44%), white (98%), married (74%), employed (69%), educated to bachelor degree level (41%), with a household income of over £60,000 (43%) and with a child under 1 (49%). Table 1 provides full demographic details.

**Table 1.** Demographic information for survey participants.

Demographic	N	%
**Age**	**151**	
18–24	4	2.6
25–30	31	20.5
31–35	66	43.7
36–40	35	23.2
41–45	15	9.9
**Marital status**	**151**	
Married	112	74.2
Single	3	2.0
Divorced	0	0
Separated	0	0
With partner	36	23.8
Prefer not to say	0	0
**Child's age**	**151**	
< 12 months	74	49
12–18 months	25	16.6
18–24 months	31	20.5
2 years +	21	13.9
Prefer not to say	0	0
**Ethnicity**	**151**	
White	148	98
Black/African/Caribbean/Black	0	0
British	1	0.7
Asian/Asian British	1	0.7
Mixed/multiple ethnic groups	1	0.7
Other ethnic group	0	0
Prefer not to say		
**Work status**	**150**	
Employed	104	69.3
Self-employed	16	10.7
Unemployed	23	15.3
Student	2	1.3
Stay at home mum	0	0
Not able to work	3	2
Other	2	1.3
Prefer not to say	0	0
**Income**	**151**	
0–£20,000	12	7.9
£20,001–£40,000	25	16.6
£40,001–£60,000	43	28.5
£60,000 +	65	43
Prefer not to say	6	4
**Education**	**151**	
No formal education	0	0
School qualifications	17	11.3
Vocational training	18	11.9
Bachelor's degree	62	41.1
Master's degree	43	28.5
Doctorate degree	7	4.6
Other	4	2.6
Prefer not to say	0	0
**Country**	**147**	
UK/Scotland/England/Wales	139	94.6
South Africa	2	3.4
The United States	5	1.4
Cayman Islands	1	0.7

#### Social networking sites usage

Participant use of SNS was extensive. The most frequently used SNS were WhatsApp (used often/a lot by 88% of respondents) and Facebook (used often/a lot by 96% of respondents) (see Table 2). The mean number of SNS used was 2.47 (range = 1–5). Most participants spent between 1 and 4 hours on SNS per day (77%) (Table 3).

**Table 2.** Social networking sites usage by site for n = 151 participants.

	Never (1)		Rarely (2)		Sometimes (3)		Often (4)		A lot (5)	
	n	%	n	%	n	%	n	%	n	%
Facebook	0	0	0	0	7	4.6	37	25	107	71
Instagram	37	25	14	9	16	11	29	19	55	36
Twitter	73	48	42	28	20	13	9	5.9	7	4.6
Linkedin	97	64	27	18	17	11	6	3.9	3	1.9
WhatsApp	7	4.6	3	1.9	8	5.2	24	16	109	72
Pinterest	28	19	64	42	48	32	10	7	1	0.6
Reddit	125	83	21	14	4	2.6	1	0.6	0	0
Google +	104	69	15	10	12	8	14	9	6	3.9
Other			2				2		9	

**Table 3.** Time spent using social networking sites (hours per day) for n = 151 participants.

How much time do you spend on social networking sites per day?													
<15 minutes		30 minutes – 1 hours		1–2 hours		3–4 hours		5–6 hours		7–8 hours		9 hours	
n	%	n	%	n	%	N	%	n	%	N	%	n	%
1	0.6	16	11	57	38	59	39	15	10	3	1.9	0	0

#### Stress, wellbeing and support

Table 4 provides descriptive statistics for model variables. SNSUN mean (67.6) and standard deviation (SD) (± 8.88) scores were moderate, given possible score ranges of 20 to 100, although scale newness restricts comparison. Cognitive needs registered highest out of the five online support sub-domains (m = 15.8, SD =  ±1.94). SWEMWBS scores were lower than UK 2011 population levels (m = 23.6, SD =  ±3.90, Health Survey England, 2011, cited in Taggart et al.,^38^) t(150) = −5.45, *p* < 0.001. PSS means (m = 40.9, SD = ± .84) are higher than in previous maternal studies,^19^ for example, Ford, 2011 (m = 36.89, SD = ± 11.68), t(150) = 5.60, *p* < 0.001.

**Table 4.** Descriptive statistics for model variables.

	Mean	SD
SNSUN	67.6	± 8.88
Affective Needs	12.7	± 2.98
Cognitive Needs	15.8	± 1.94
Personal Needs	10.7	± 3.43
Social Needs	13.5	± 2.73
Diversion Needs	14.8	± 2.44
PSS	40.9	± 8.84
SWEMWBS	21.9	± 3.76

PSS: Parental Stress Scale; SD: standard deviation; SNSUN: Social Networking Site Usage and Needs Scale; SWEMWBS: Short Warwick Edinburgh Wellbeing Scale.

### Phase 2: Relationships between variables

There was a significant positive correlation between income and mental wellbeing such that women with higher household incomes recorded higher mental wellbeing r(151) = 0.17, *p* = 0.035 (confidence interval (CI) 0.01, 0.33). There was also a significant positive correlation between education and parental stress such that more highly educated participants perceived higher parental stress r(151) = 0.24, *p* = 0.03 (CI 0.08, 0.38).

SNS usage frequency was not significantly correlated with either parental stress or mental wellbeing. There was a significant positive correlation between the SNSUN ‘diversion’ sub-domain and parental stress r(151) = 0.17, *p* = 0.032 such that people using social media for diversion needs (e.g. escape) perceived more stress. There was a significant negative correlation between mental wellbeing and parental stress such that mothers with higher mental wellbeing scores perceived less parental stress r(151) = −.60, *p* < 0.001 (CI −0.69, −0.49).

Non-significant small effect sizes demonstrate associations between both the perceived value and the time spent on SNS with parental stress. Additionally, non-significant small effect sizes highlight associations between both the perceived value of SNS and the number of SNS used with mental wellbeing.

### Phase 3: Causal mediation analysis for overall online support

The direct effect of online support needs on parental stress was non-significant and explained 2% of the variance. The direct effect of online support needs on mental wellbeing was non-significant and explained 1% of the variance. The indirect effect of online support needs on parental stress through mental wellbeing explained 37% of the variance. There was a non-significant positive effect of online support needs on parental stress and a significant negative effect of mental wellbeing on parental stress. The non-significant regression weights for online support needs reduced from Step 1 to 3 confirming a non-significant partial mediation.

**Figure 1.**
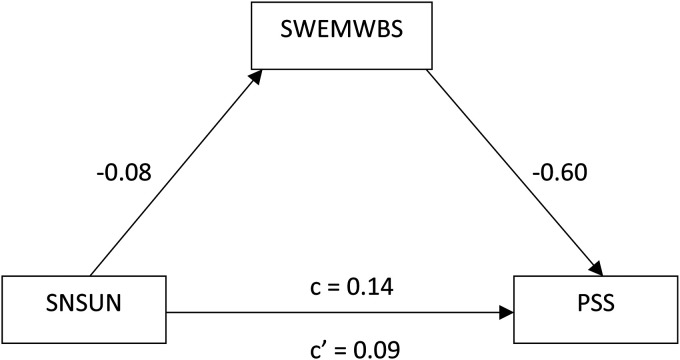
Causal mediation diagram.

The bootstrapped standardised coefficient for the indirect effect between online support needs and mental wellbeing was β 0.05 (95% CI −0.04 to 0.13) confirming the non-significant partially mediated effect. Specifically, the causal mediation analysis confirmed the presence of small positive non-significant direct and indirect effects of online support on parental stress as outlined in the mediation model in Figure 1. Table 5 details full results.

**Table 5.** Causal mediation results.

	β	SE	F (df)	T	*P*	r^2^	CI
1. Direct effect							
SNSUN*PSS	0.14	0.08	3.05 (1149)	1.75	0.083	0.02	−0.17 to 0.30
2. Direct effect							
SNSUN*SWEMWBS	−0.08	0.03	1.01 (1149)	−1.01	0.316	0.01	−0.10 to 0.03
3. Indirect effects on PSS			44.35 (2148)			0.37	
SNSUN	0.09	0.07		1.42	0.158		−0.04 to 0.22
SWEMWBS	−0.60	0.15		−9.16	<.001		−1.70 to −1.09
Bootstrapping	0.05	0.04				0.37	−0.04 to 0.13

β: standardised beta; 95% CI: 95% confidence interval; PSS: Parental Stress Scale; SE: standard error; SNSUN: Social Networking Site Usage and Needs Scale; SWEMWBS: Short Warwick Edinburgh Wellbeing Scale .

### Phase 4: Causal mediation analysis at SNSUN sub-domain level

Online support sub-domain mediation analyses (Table 6) revealed non-significant partial mediation. Effect size variation highlighted affective and diversion needs as having the strongest positive direct and indirect effects on parental stress.

**Table 6.** Causal mediation results at SNSUN sub-domain level.

SNSUN sub-domain	Direct effect on PSS		Direct effect on SWEMWBS		Indirect effect		Bootstrapped indirect effect	
	β	SE	β	SE	Β	SE	β	CI
Affective	0.16	0.24	−0.08	0.10	0.11	−0.60	0.05	−0.06 0.14
Social	0.11	0.27	−0.03	0.11	0.09	0.21	0.02	−0.07 0.13
Diversion	0.18*	0.30	−0.03	0.13	0.16*	0.24	0.02	−0.09 0.12
Personal	−0.02	0.21	−0.06	0.09	−0.06	0.17	0.04	−0.06 0.14
Cognitive	0.07	0.37	−0.05	0.16	0.04	0.30	0.03	−0.08 0.13

*p < 0.05.

β: standardised beta; 95% CI: 95% confidence interval; PSS: Parental Stress Scale; SE: standard error; SNSUN: Social Networking Site Usage and Needs Scale; SWEMWBS: Short Warwick Edinburgh Wellbeing Scale.

## Qualitative results

**Figure 2.**
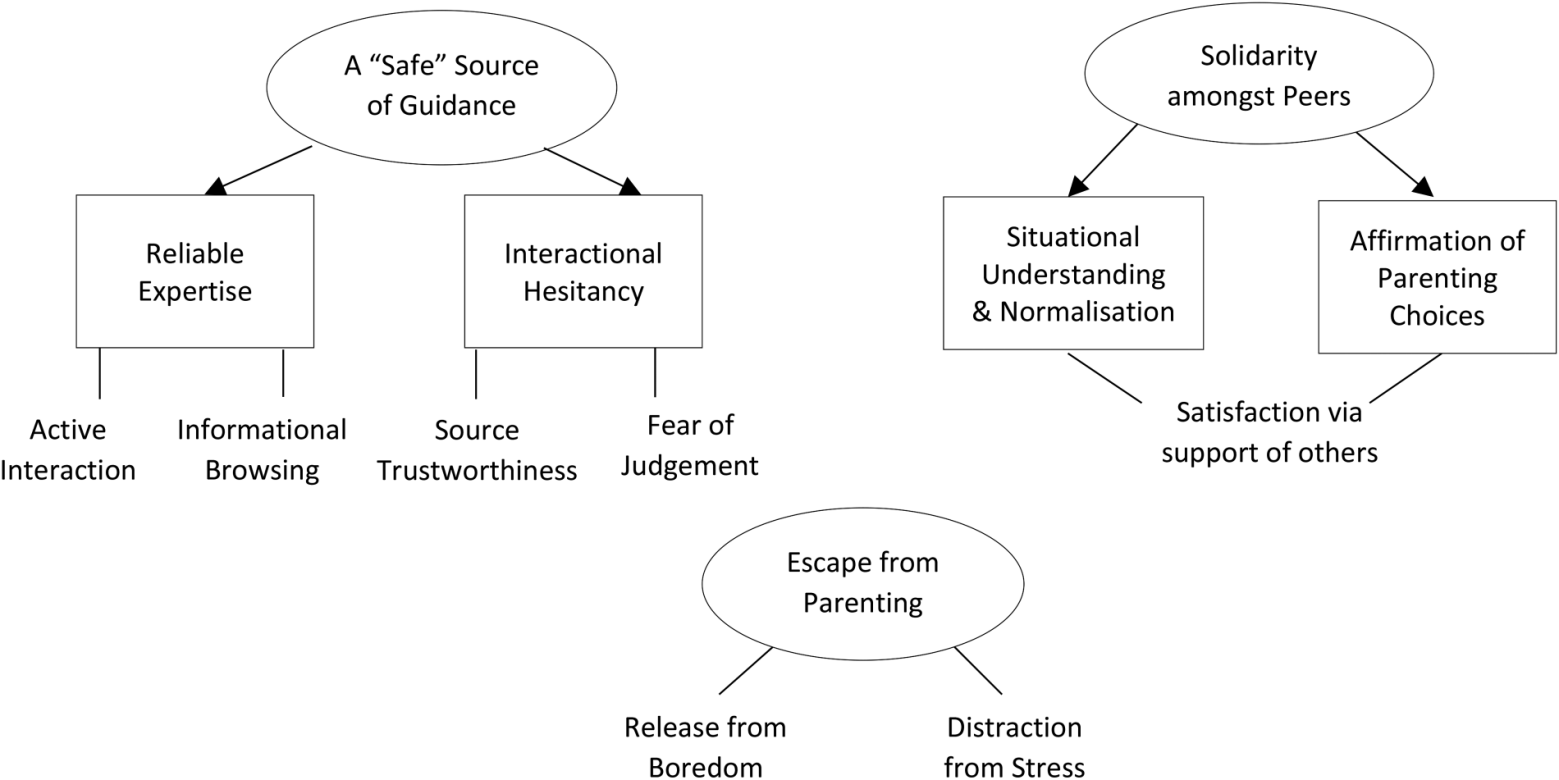
Thematic map.

The open-text response rate for each of the questions was high (Table 7) with sufficient detail to facilitate analysis. Longer responses elaborated shorter responses with a similar focus, making generated themes representative of the whole cohort. Responses indicate that participants interact online with multiple contacts (friends, family, peers and professionals) at an individual and group level. Generated themes outline how online support can influence wellbeing by providing a ‘safe’ source of guidance, solidarity amongst peers and escape from parenting. The thematic map in Figure 2 details sub-thematic connections.

**Table 7.** Qualitative open-text response summary.

Open-text survey statement	Proportion of participants entering responses (n (%))	Proportion of responses >50 characters (n (%))
Please could you describe your online interactions, e.g., what people/groups do you connect with?	144 (95)	114 (75)
Thinking about the interactions described above, what are you looking to get out of these interactions and are the interactions generally useful?	143 (94)	90 (60)
If you had parenting experience prior to COVID-19, how does your online social interaction during COVID-19 compare with your online social interaction prior to COVID-19?	132 (87)	62 (41)
Please could you give an example(s) of a common parenting stress for you?	142 (94)	97 (64)
Thinking of the above example(s), how does it/they impact your daily life? What actions (if any) do you normally take to relieve the stress?	140 (93)	115 (76)
Please could you describe your general mood and how it impacts on your daily life?	139 (92)	104 (69)

### A ‘safe’ source of guidance

Online support was commonly used to aid problem resolution by providing reliable expertise in a space that took account of some interactional hesitancy by cautious participants. Selected sites offered trustworthy support and spaces to passively garner support via published discussions, without any need to actively participate (out of distrust or fear of judgement).

#### Reliable expertise

The majority of participants highlighted online spaces as sources of trustworthy advice when dealing with parenting challenges either through active problem resolution or passive browsing for ideas. The participants below actively seek out online interactions to gain practical guidance to cope with high levels of parental stress and mental wellbeing struggles:

‘It has provided me with invaluable support and advice that has enabled me to continue to breastfeed through some very challenging times.’ (783846/a)

‘Try to actively educate myself on helping children deal with their emotions to ease meltdowns in the future and prevent such stressful situations.’ (922880/e)

In contrast to this active problem resolution, mothers also commonly talk of routine browsing for helpful information. This passively sourced information also improves participant stress management and coping:

‘I'm always open to new activities for kids or items for working with behaviors.’ (224225/b)

‘Learn useful hints and tips from others problems or new places to visit etc. ‘(120514/b)

One point of caution, however, is that some participants discuss their increased dependency on online information due to COVID-19. For some participants, COVID-19 restrictions have created an additional need for inspirational and logistical information through online spaces, which may not reflect normal activity:

‘I use Facebook for lots of ideas of activities to do with my toddler, in a way that I didn't use it for this purpose pre COVID. (722611/c)

#### Interactional hesitancy

Whilst the majority of participants describe a range of common online communications, a minority of mothers emphasise their hesitancy around online interactions. This respondent values the informative nature of online support but is cautious about source trustworthiness:

‘I'm careful about the source as most sites cannot be trusted’. (198824/b)

Similar hesitancy is shown in this next example which highlights anonymously garnering knowledge without any pressure of engagement for fear of judgement.

‘Mostly browse or search for specific things. Tend not to post as nervous of trolling and lack of privacy. (223194/b)

In summary, online support sites are considered by most to be valuable sources of predominantly reliable advice, delivered in spaces providing anonymity for mothers.

### Solidarity amongst peers

A second common theme was the peer solidarity offered through the use of online support.

#### Affirmation of parenting choices

Online groups are accessed by some participants as a means to validate their parenting decisions. The participant below utilises online peer support to build confidence in her parenting actions. The example shows a mother struggling with uncertainty over her parenting choices and offers hints of underlying tensions caused by societal norms:

‘I seek approval that what I am doing is okay. That it's considered the right thing. Even though I know these strangers know no more than me it is nice to know others view your parenting as good. (193890/b)

Validation of parenting-related perspectives is similarly captured by the response below:

‘quite a few of the people I know support sleep training and I do not. Reading the posts helps me understand why it isn't something I support and not get swayed by others advice in real life.’ (285726/a)

The emotionally charged nature of parenting decisions can lead to uncertainty and hesitancy for these mothers. Their use of online peer support provides a mechanism for increasing confidence by validating their choices, even when they conflict with social norms.

#### Situational understanding and normalisation

Most mothers highlight their use of online support as a means to connect with people experiencing similar circumstances. This next participant seeks online peer support to gain reassurance about the normality of her challenges:

‘I speak to other parents in a similar situation to try and relieve stress - realising I'm not the only one is very reassuring’. (752608/e)

Such interactions improve participant coping, as illustrated below. This highlights how online peer solidarity is commonly described as involving both giving and receiving support:

‘There is always another parent on there with a similar story to remind me that one cannot give from an empty cup. That tends to spur me into action’. (331822/f)

Offering online support is a valuable means of connection for some. This participant indicates that COVID-19 has increased her reliance on online peer interactions:

‘I probably spend more time supporting others now than before. I rely more on these interactions to make me feel connected, especially during lockdown’. (420508/c)

However, for some mothers, online connection is less helpful but driven by COVID-19 restrictions:

Pretty low (general mood). But it has been improving since we have been able to get out to join a real-life baby group. I don't want to talk to people online- I like to see real people! (193890/f)

In summary, online support provides valued peer connection to validate parenting choices and normalise challenging circumstances. However, for some participants, online support remains a poor replacement for real-life contact.

### Escape from parenting

Online support provides a source of distraction. These mothers seek escape from monotonous and relentless experiences:

If I'm bored, I will browse my nappy groups and news feed for some superficial chat (331822/b)

I zone out on my phone as it's the only break/ social interaction/ change of 'scene' I can get. (722464/e)

However, such mindless diversion has negative connotations for some:

All useful except my reliance on mindless scrolling. Could waste hours on it! (935698/b)

In summary, online support provides a source of escape from the boredom and stresses of parenting. However, this function is often regarded negatively.

## Discussion

Within this study, mixed-method findings combined to illuminate the complex and pressurised perinatal experience of some women. There was a positive relationship between participants’ online support usage and needs, predominantly sought through Facebook and WhatsApp, and their perceptions of parental stress. This correlation was partially mediated through mental wellbeing, suggesting that the positive relationship between social media needs and stress was reduced for mothers with higher mental wellbeing. Mothers perceiving greater online support value scored lower on mental wellbeing. The non-significant effect size variation of online social support at sub-domain level highlighted affective and diversion needs as having the strongest positive direct and indirect effects on parental stress, indicating a social network usage and needs focus on emotional aspects of social support.

Although non-significant results and small effect sizes reduced relationship certainty, qualitative insights aided interpretation of quantitative relationships, demonstrating a focused use of online support during periods of high parental stress and maternal wellbeing struggles. Participants described a turbulent parenting journey, with fluctuating uncertainty and parental stress, with the sub-theme of interaction hesitancy further illustrating participant vulnerability and isolation. SNS usage and needs offered participants a timely coping strategy by providing a safe source of peer expertise, affirmation of parenting choices and the normalisation of parenting challenges when most needed. The use of online support as a coping strategy is supported by previous research, which highlights maternal use of online support to offset perceived limitations of health professional provision.^4,5,49^

Taken together, these mixed-method findings suggest that mothers experiencing high stress and low mood, value the online support they utilise as part of their stress coping strategies and suggest that the interactions offer an element of support not accessible elsewhere. Notably, participants reported higher stress and lower mental wellbeing than pre-COVID comparators, supporting recent maternal mental health COVID findings^7,8^ and WHO strategies.^1^

The small size and non-significance of correlational results combined with the cross-sectional study design, which does not allow us to determine the direction of these effects, limit our causal understanding. This prompts exploration of alternative interpretations. Whilst poorer wellbeing provides a plausible explanation for valuing online support more in times of perceived stress, sample mothers may not explicitly initiate SNS activity with the intention of seeking support for their parental stress. The significant relationship between SNSUN diversion and parental stress, for example, illustrates how the use of social media can be an escape from parenting stresses.

Additionally, qualitative ‘interactional hesitancy’ sub-themes discuss a fear of judgement and of untrustworthy information, intimating online conflict which could potentially induce stress and account for positive correlations. However, given the dominance within qualitative themes of online support as a helpful and reassuring experience, such stress-inducing interactions could only partially explain the positive relationship. Nevertheless, the cautious approach to online contact indicated by some mothers suggests limited interaction quality which could plausibly explain small associations. However, qualitative themes also highlight SNS activity variation often shaped by current stress appraisal, underlining the complexity of the stress-support relationship.^13^ Our interpretations of online coping strategy efficacy and wellbeing improvement opportunities must therefore remain cautious without longitudinal insights.

### Study limitations

Whilst this study benefited from the use of mixed-methods, limitations are evident. Larger sample sizes, longitudinal designs or semi-structured interviews would offer better opportunities to explore causality and participant perceptions and experiences. Despite survey design measures encouraging response description, study interpretation is restricted by the chosen format and the inability to explore comments, and time sequences in more depth, for example by capturing potential variation of SNS usage purpose.

Additionally, recruitment procedures may have unintentionally constrained gathered participant experiences to parenting forums and stress-related interactions. Facebook focused social media adverts, for example, will have inevitably shaped gathered insights to those individuals who use, or perhaps know people who use Facebook, and therefore additional studies focused on alternative SNS platforms such as Instagram may provide additional narratives. Furthermore, survey questions directing participants to consider their parenting stresses may have unintentionally constrained comments, with an interview format perhaps providing greater discussion scope. Interviews could also clarify the degree and nature of COVID-19's impact on online support activity.

A further study limitation is the participant demographic. Despite extensive recruitment strategies, participants are predominantly white, married, highly educated and with a large household income. Whilst social media sampling methods aimed to maximise online support insights, we do not know about perceptions of mothers without online support. Collectively, these factors demonstrate a need for further in-depth research that explores the potential impact of diverse social backgrounds (e.g. ethnicity), perinatal stages (e.g. initial motherhood uncertainty), parenting styles (e.g. sleep training) and mothers without online support.

Perinatal online support, whilst no different from face-to-face support in some respects, is characterised by greater variation in quality and accessibility. Peer opinion and empathy combined with the possibility of anonymity, make online spaces a valued means of support worthy of attention, particularly where there is a focus on high-quality content. Further research could help to understand how best to increase stress-coping efficacy and facilitate broader wellbeing interventions within online spaces.

This study increases understanding of maternal online interactions and highlights opportunities to improve perinatal anxiety coping strategies. Health professionals working with new mothers should be aware of hidden vulnerabilities that perinatal mothers may be reluctant to share as they navigate complex and fluctuating parental stress. Online support offers an anonymity not possible face-to-face providing a safe and reliable source of immediate validation, solidarity and guidance during periods of peak stress. Midwives and health visitors, restricted as they are with limited resources and availability, should be aware of likely hidden maternal vulnerabilities and of the potential for an associated prolific use of SNS to provide support outside the clinic environment. Health professionals could guide mothers to reliable evidence-based sources, for example, Parent Club resources, The Breastfeeding Network, the BabyBuddy App from Best Beginnings UK, as well as local trusted parenting support networks. Finally, a broader focus on facilitating quality online relationships for parents may be increasingly important to improve long-term maternal and child health.

## Appendix

**Appendix A.** Recruitment breakdown

Group name	Facebook page
Active Stirling Buggy Walking Group	https://www.facebook.com/walkforthbumpsandbabies
The Daisy Foundation (Stirling Branch) birthing and parenting group	https://www.facebook.com/groups/562900357159643/
Association of Breastfeeding Mothers	https://www.facebook.com/AssocBfMothers
Studio 63, Killearn. Parent and Children exercise classes	https://www.facebook.com/communityfitness63

**Appendix B.** Online survey components

Parental Stress Scale (PSS) Berry & Jones (1995)		
The following statements describe feelings and perceptions about the experience of being a parent. Think of each of the items in terms of how your relationship with your child or children typically is. Please indicate the degree to which you agree or disagree with the following items by ticking the box that is most relevant to you.		
PSS Items 1, 2, 5, 6, 7, 8, 17 and 18 are reverse scored as follows: (1 = 5) (2 = 4) (3 = 3) (4 = 2) (5 = 1) (higher score = higher stress)	1. I am happy in my role as a parent 2. There is little or nothing I wouldn't do for my child(ren) if it was necessary. 3. Caring for my child(ren) sometimes takes more time and energy than I have to give. 4. I sometimes worry whether I am doing enough for my child(ren). 5. I feel close to my child(ren). 6. I enjoy spending time with my child(ren). 7. My child(ren) is an important source of affection for me. 8. Having child(ren) gives me a more certain and optimistic view for the future. 9. The major source of stress in my life is my child(ren). 10. Having child(ren) leaves little time and flexibility in my life. 11. Having child(ren) has been a financial burden. 12. It is difficult to balance different responsibilities because of my child(ren). 13. The behaviour of my child(ren) is often embarrassing or stressful to me. 14. If I had it to do over again, I might decide not to have child(ren). 15. I feel overwhelmed by the responsibility of being a parent. 16. Having child(ren) has meant having too few choices and too little control over my life. 17. I am satisfied as a parent. 18. I find my child(ren) enjoyable.	Scoring Tick the relevant box 1 = Strongly disagree 2 = Disagree 3 = Undecided 4 = Agree 5 = Strongly agree Possible scoring range from 18 to 90

**Table:**

Qualitative open-text questions
Please could you give an example(s) of a common parenting stress for you?
Thinking of the above example(s), how does it/they impact your daily life? What actions (if any) do you normally take to relieve the stress?

**Table:**

Online social support (using Social Networking Sites Usage and Needs Scale (SNSUN))			
Below are some questions about your use of social networking sites			
	How often do you use the following social networking sites? Facebook Instagram Twitter Linkedin Whatsapp Pinterest Reddit Google + **Other:** If you use other sites, apps or services, please write them in and rate how often you use them		Scoring: Tick the box that best describes your experience of each over the last 2 weeks Never, rarely, sometimes, often, a lot or does not apply to me
Thinking of the above sites, please answer the following questions:			
SNSUN Statements highlighted in blue were removed following pilot feedback	How often do you use social networking sites?		Never, rarely, sometimes, often, a lot or does not apply to me
	How many social networking sites do you actively use?		1, 2, 3, 4, 5, more than 5 or does not apply to me
	How often do you check your social networking sites per day?		Less than once a day, 1–2 times per day, 3–4 times per day, 5–6 times per day, 7–8 times per day, 9 + times per day, on every notification or does not apply to me
	How much time do you spend on social networking sites per day?		Less than 15 minutes, 30 minutes–1 hour, 1–2 hours, 3–4 hours, 5–6 hours, 7–8 hours, 9 hours + or does not apply to me
Below are some questions about your use of social networking sites. Please tick the box that is most relevant for you.			
SNSUN The wording of questions in red were amended slightly to make them more context specific High score = high usage	Diversion	Using social networking sites helps me to feel less lonely I use social networking sites to pass time when I’m bored Social networking sites let me escape my worries I start using social networking sites when I have nothing better to do	Scoring: Tick the box 1 = Strongly disagree, 2 = Disagree, 3 = Undecided, 4 = Agree, 5 = Strongly agree or 0 = does not apply to me Possible scoring range from 0 to 100
	Cognitive needs	Social networking sites help me with parenting-related research Social networking sites help me find and connect to online businesses Social networking sites help me to gain knowledge Social networking sites give me information about others	
	Affective needs	Using social networking sites is something I routinely do when I am online Social networking sites help me express my emotions to others easily Social networking sites allow me to develop my relationships I use social networking sites to talk about my problems and get advice	
	Personal integrative needs	Social networking sites are important for my self-image Social networking sites portray an image of me to others People can use social networking sites to judge me I use social networking sites to gain favourable approval amongst friends	
	Social integrative needs	Social networking sites allow me to communicate with my friends Social networking sites allow me to stay in touch with my family Social networking sites enable me to find more interesting people than in real-life Social networking sites enable me to get through to someone who is hard to reach	

**Table:**

Qualitative open-text questions
Please could you describe your online interactions, e.g., what people/groups do you connect with?
Thinking about the interactions described above, what are you looking to get out of these interactions and are the interactions generally useful?
If you had parenting experience prior to COVID-19, how does your online social interaction during COVID-19 compare with your online social interaction prior to COVID-19?

**Table:**

Short Warwick–Edinburgh Mental Wellbeing Scale (SWEMWBS) © NHS Health Scotland, University of Warwick and University of Edinburgh, 2007, all rights reserved.		
Below are some questions about feelings and thoughts. Please tick the box that best describes your experience of each over the last 2 weeks		
Short Warwick- Edinburgh Mental Wellbeing Scale SWEMWBS Higher score = better wellbeing	I’ve been feeling optimistic about the future I’ve been feeling useful I’ve been feeling relaxed I’ve been dealing with problems well I’ve been thinking clearly I’ve been feeling close to other people I’ve been able to make up my own mind about things	Scoring: Tick the box that best describes your experience of each over the last two weeks 1 = None of the time, 2 = rarely, 3 = some of the time, 4 = often, 5 = all of the time Possible scoring ranges from 7 to 35

**Table:**

Qualitative open-text questions
Please could you describe your general mood and how it impacts on your daily life?

End of Survey
